# 28 Osteoarticular manifestations simulating JIA in acute leukemia: case report and literature review

**DOI:** 10.1093/rheumatology/keac496.024

**Published:** 2022-10-07

**Authors:** N Benmouffok, F. Z Boudouaa, S Tari, R Nemmar, A Boulainine, S Rouabeh, S Ouatah, Z Zeroual

**Affiliations:** Pediatric Hematology-Oncology Unit, Pediatrics Service A. Pr Z. Zeroual, UHC Nafissa Hamoud Algers

## Abstract

**Background:**

The osteoarticular manifestations are proven during hematological malignancies in children, they can be the revealing symptom of the disease, and they may simulate a full clinical picture of Juvenile Idiopathic Arthritis (JIA), especially if no blasts are found in blood smear. Some therapies are common to both diseases, which commonly delay diagnosis for a couple of months and worsens the prognosis of these malignancies. We report three observations of patients treated for JIA which turned out to be acute leukaemia.

**Clinical caises:**

The first patient is a 7-year-old male followed in pediatric rheumatology consultation for a monoarticular onset JIA. Since the patient did not respond to indomethacin, a bone marrow examination (BME) was performed before corticosteroid therapy (CST), no blasts were found. The patient received 01 month of CST which led to the amendment of clinical and biological signs, however, joint pain and bicytopenia reappeared after we started CST reduction. Blood and blood marrow smear were performed, leading to the diagnosis of acute B-lymphoblastic leukaemia.

The second patient is a 4 years old male admitted for exploration of polyarthritis with cervical lymphadenopathy, the initial assessment allowed the diagnosis of JIA with polyarticular onset. Indomethacin is prescribed, without any improvement. Radiological assessment was performed, as well as a complete blood count (CBC) which found neutropenia with lymphopenia, and blasts were discovered in the second BME.

The third patient is a 6-year-old female admitted for exploration of arthralgia and pathological fractures. Radiological signs in favor of leukaemia and normochromic aregenerative normocytic anaemia were revealed, allowing the diagnosis of ALL B to be made after several consultations in rheumatology.

**Discussion:**

Joint damage during leukaemia, called leukaemic arthritis, is frequent, complicating 12% to65% of pediatric leukaemia, most often ALL. It is linked to specific damage by leukaemic infiltration of the synovium or less likely to a synovial reaction to adjacent periosteal or capsular infiltration. In JIA there are incomplete or atypical forms, the differential diagnoses to be sought are numerous and depend on age, personal and family anamnesis and clinical, biological, hematological and radiological signs. The dread of a haematological malignancy must constantly be sought and excluded before the use of CST, which is one of the therapeutic weapons in ALL, thus causing the blasts to disappear and delaying the diagnosis.

**Conclusion:**

The diagnosis of JIA must be made with caution, raise the possibility of haematological malignancy in children in the absence of response to usual treatments, in the presence of systemic signs such as fever or a persistent biological syndrome or atypical radiological lesions, hence the correct analysis of the CBC and the blood and marrow smears is clearly important.

